# Association between Dietary Hardness and Cognitive Dysfunction among Japanese Men in Their 60s: A Cross-Sectional Study

**DOI:** 10.3390/nu15112485

**Published:** 2023-05-26

**Authors:** Aya Fujiwara, Ami Fukunaga, Kentaro Murakami, Yosuke Inoue, Tohru Nakagawa, Shuichiro Yamamoto, Maki Konishi, Tetsuya Mizoue

**Affiliations:** 1 Department of Epidemiology and Prevention, Center for Clinical Sciences, National Center for Global Health and Medicine, 1-21-1 Toyama, Shinjuku-ku, Tokyo 162-8655, Japan; 2 Department of Social and Preventive Epidemiology, School of Public Health, University of Tokyo, 7-3-1 Hongo, Bunkyo-ku, Tokyo 113-0033, Japan; 3 Department of Nutritional Epidemiology and Shokuiku, National Institutes of Biomedical Innovation, Health and Nutrition, 1-23-1 Toyama, Shinjuku-ku, Tokyo 162-8636, Japan; 4 Hitachi Health Care Center, Hitachi, Ltd., 4-3-16 Osecho, Hitachi-shi 317-0076, Ibaraki, Japan

**Keywords:** hardness, mastication, cognitive impairment, older adults, Japan

## Abstract

We aimed to examine the cross-sectional association between dietary hardness and cognitive dysfunction among Japanese men in their 60s. Participants were 1494 men aged 60–69 years from the baseline survey of Hitachi Health Study II (2017–2020). Dietary hardness was defined as an estimate of masticatory muscle activity involved in consuming solid foods. Habitual intake of these foods was assessed using a brief-type, self-administered diet history questionnaire. Cognitive dysfunction was defined as a score ≤ 13 points on the test battery for screening for Alzheimer’s disease (MSP-1100). The mean (SD) age of participants was 63.5 (3.5) years. The prevalence of cognitive dysfunction was 7.5%. The ORs (95% CIs) for cognitive dysfunction in the second and third tertiles were: 0.77 (0.47, 1.26) and 0.87 (0.54, 1.41), respectively, after adjustment for socio-demographic factors (*p* for trend = 0.73). After further adjustment for protective nutrient intake against cognitive dysfunction, the corresponding figures were 0.72 (0.43, 1.21) and 0.79 (0.43, 1.46), respectively (*p* for trend = 0.57). Dietary hardness was not associated with the prevalence of cognitive dysfunction among Japanese men in their 60s. Future prospective studies are necessary to investigate the association between dietary hardness estimated by a validated questionnaire and cognitive dysfunctions.

## 1. Introduction

With a global increase in the ageing population, individuals with cognitive dysfunction and impairment have emerged. More than 55 million individuals were estimated to live with dementia by 2022, with nearly 10 million new cases annually [1], and this number is projected to reach 78 million by 2030 [2]. Dementia is the seventh leading cause of death and a major cause of disability and dependency among older adults [1]. Given the lack of curative treatment for dementia [3], more efforts are necessary to identify modifiable factors to prevent or delay the onset and progression of cognitive dysfunction.

Although several dietary components, such as n-3 polyunsaturated fatty acid (PUFA), antioxidants, vitamin D, vitamin B including folate, fruits and vegetables, fish, and the Mediterranean diet, have been extensively investigated [4,5], a new line of study has recently focused on the influence of hardness, another aspect of a diet, on cognitive dysfunction. For example, animal studies [6,7,8] have suggested that consuming a soft diet reduces masticatory sensory input and decreases synaptic formation in the cerebral cortex and neurogenesis in the hippocampus. Given that human experimental studies have suggested that masticatory activity increases blood flow and oxygenation in a wide range of cerebral regions related to learning and memory processing [9], it can be hypothesised that a hard diet that induces masticatory activity may improve cognitive function and thus prevent the onset of cognitive dysfunction.

The previous cross-sectional [10,11,12,13] and prospective [14,15] epidemiological studies in this field have mainly examined the influence of masticatory ability and oral health status on cognitive function rather than that of hardness of the habitual diet. Higher mastication ability evaluated by gum chewing was associated with higher cognitive function among older Japanese [10] and older Korean women [11]. Self-assessed chewing ability was positively associated with better cognitive function among middle-aged to older adults from 14 European countries [12]. Meanwhile, self-assessed chewing difficulty was associated with the higher prevalence and onset of cognitive impairment in older Swedish [13] and middle-aged to older Koreans [14], respectively, and a higher onset of dementia in older Japanese [15]. Meanwhile, only Okubo et al. [16] examined the cross-sectional association between hardness of the habitual diet and cognitive function among 635 Japanese community dwellers aged 69–71 years. In their study, the positive association observed in the models adjusted for sociodemographic variables disappeared after further adjustment for the intake of nutrients protective against cognitive function [16]. Given that understanding the association between the hardness of habitual diet and cognitive dysfunction may offer further insights into the prevention of cognitive dysfunction and dementia, more studies should be conducted on this issue.

Hence, the present study aimed to examine the association between the hardness of the habitual diet and cognitive dysfunction among Japanese men in their 60s based on a cross-sectional design. We hypothesised that higher dietary hardness would be associated with a lower prevalence of cognitive dysfunction.

## 2. Materials and Methods

### 2.1. Study Design and Participants

**Figure 1:**
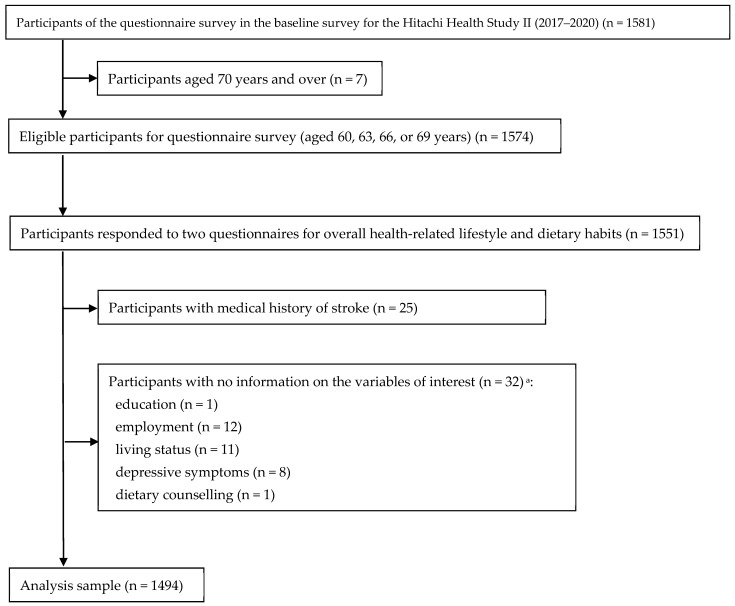
Flowchart of participants included in the present analysis. ^a^ Some participants without several variables.

This cross-sectional study was based on data from a baseline survey of Hitachi Health Study II. The Hitachi Health Study II is an ongoing prospective study conducted on current and retired employees and their spouses at Hitachi, Ltd., Ibaraki, Japan, a manufacturing company in the Ibaraki Prefecture. Data collection for the baseline survey was conducted as part of the health check-up from April 2017 to March 2020. Of those who underwent health check-ups, individuals aged 60 years and over (as of the 31st of March each year) and screened for their cognitive function were instructed to participate in the baseline survey. We further instructed participants aged 60, 63, 66, or 69 years to fill out two questionnaires for overall health-related lifestyle and dietary habits on the day of the health check-up. Because of the aim of the present study, we considered those who underwent cognitive function screening and questionnaire surveys as the target of the present analysis (*n* = 1581). No women were included because of the small proportion of the original sample (10.4%). For the present analysis, after excluding those aged 70 years and over (*n* = 7), we included participants who completed both questionnaire surveys (*n* = 1551). We further excluded those with a medical history of stroke (*n* = 25) and those with no information on the variables of interest (*n* = 32). In total, 1494 men were included in the final analysis (Figure 1).

This study was conducted in accordance with the guidelines of the Declaration of Helsinki. All procedures involving human subjects were approved by the Ethics Committee of the National Center for Global Health and Medicine (approval number: NCGM-G-002208) and Hitachi Health Care Center. Written informed consent was obtained from all participants before participating in the present study.

### 2.2. Estimation of Dietary Hardness

Dietary hardness in the present study was defined as an estimate of the masticatory muscle activity required for the consumption of the habitual diet, according to previous studies [16,17,18,19]. Habitual dietary intake was assessed using a previously validated, brief-type, self-administered diet history questionnaire (BDHQ) [20,21]. The BDHQ estimates daily intake of 58 items (consisting of 38 foods, 12 beverages, and 8 seasonings) during the preceding month, which were commonly consumed in Japan [20]. The BDHQ is a fixed-portion size questionnaire that asks about the frequency of selected food consumption but not portion size. Intakes of energy and selected nutrients were calculated using an ad hoc computer algorithm for the BDHQ [21], according to the Standard Tables of Food Composition in Japan, 2010 [22]. The BDHQ has a satisfactory ranking ability of energy-adjusted dietary intake using the density method in Japanese men aged 32–76 years [20,21]. The median Spearman’s correlation coefficient with 16-day dietary records was 0.48 (interquartile range, 0.33–0.56) for food groups [20], while Pearson’s correlation coefficient was 0.56 (interquartile range, 0.41–0.63) for nutrients [21].

Subsequently, the dietary hardness of each participant’s habitual dietary intake (mV·s/day) was calculated by summing the products of the hardness of each food item in the BDHQ (mV·s/cm^3^) and its volume consumed (cm^3^/day) [16,17]. Briefly, to estimate the hardness of each food item (mV·s/cm^3^), 34 out of 38 food items in the BDHQ were directly matched to an equivalent food item for which information on masticatory muscle activity (mV·s/2.197 cm^3^) was available from Yanagisawa et al. [19] and then divided by 2.197 [16]. The corresponding values for similar food items were used as proxies for the remaining four food items, whereas the hardness of beverages and seasonings was not estimated [16]. Given the great influence of cooking methods on the hardness of vegetables [19], the observed ratio of consumption of raw/cooked form (S. Sasaki, unpublished observations, 2006) was considered as much as possible in the matching procedure [16,17]. Food volume consumed (cm^3^/day) was estimated based on the weight in grams (g/day) assessed using the BDHQ by assuming that the density for all foods was 1 (g/cm^3^) [16,17].

To consider differences in dietary intake due to varying body sizes and energy requirements and to attenuate the influence of misreporting and a high correlation between energy intake and the crude estimate of dietary hardness, dietary hardness and dietary intake were energy-adjusted using the density methods [23]. Because of the higher proportion of alcohol consumers in the present study (75.2%) than in a previous study (37.0%) [16] and the non-contribution of fluids, including alcoholic beverages, to the estimation of dietary hardness, energy-adjusted dietary hardness was provided as the value per 1000 kcal of energy intake from solid foods (i.e., foods and seasonings). Energy-adjusted nutrient intake is presented as units/1000 kcal of total energy intake.

### 2.3. Assessment of Cognitive Dysfunction

Cognitive dysfunction was assessed using a computerised test battery for screening individuals at risk of Alzheimer’s disease (MSP-1100, Nihon Kohden Corporation, Tokyo, Japan) [24]. Briefly, the test battery was developed based on the revised version of Hasegawa’s Dementia Scale [25]. It consisted of 4 tasks for examining temporal memory (3 items), temporal orientation (4 items), three-dimensional visual-spatial perception (2 items), and short-term memory (3 items) of participants [24]. Each item was scored as 1 (for the former 3 tasks) or 2 (for the latter 1 task) points for each correct response. The score ranged from 0 to 15 points, with a higher score indicating cognitive improvement. According to previous studies [24,26], participants who scored ≤ 13 points on the test battery were defined as having cognitive dysfunction.

### 2.4. Assessment of Covariates

For the assessment of covariates, we referred to the participants’ health check-up data, including anthropometric and biochemical measurements and information on the history of diseases, and the overall health-related lifestyle questionnaire. Body height and weight were measured to the nearest 0.1 kg and 0.1 cm, respectively, while the participants wore light clothes and no shoes. Body mass index (BMI, kg/m^2^) was calculated as body weight divided by height squared. Blood pressure was measured using an automatic sphygmomanometer. Fasting plasma glucose (FPG) level was measured using the glucose oxidase enzyme electrode method (A&T, Tokyo, Japan), and haemoglobin A1c (HbA1c) level was measured using high-performance liquid chromatography (HLC723-G9, TOSOH, Tokyo, Japan). Alcohol consumption was calculated based on information on the frequency and amount of alcohol consumption collected during the health check-up. Hypertension was defined as present when participants had systolic blood pressure ≥ 140 mmHg, diastolic blood pressure ≥ 90 mmHg, and/or self-reported medication use for hypertension [27]. Diabetes was defined as present when participants had FPG level ≥ 126 mg/dL, HbA1c level ≥ 6.5%, and/or self-reported medication use for diabetes [28]. Depressive symptoms were measured using the Japanese version of the short version of the Center for Epidemiologic Studies Depression Scale, which consists of 11 of the original 20 items [29,30,31]. In line with a previous study that used arithmetic conversion to define a cut-off score, those who scored ≥ 9 points were defined as having depressive symptoms [32].

### 2.5. Statistical Analyses

Descriptive data are presented as means and standard deviations (SDs) for continuous variables or numbers and percentages of participants for categorical variables. Energy-adjusted dietary hardness (mV·s/1000 kcal) was categorised into tertiles and used to compare the selected characteristics of the participants.

Odds ratios (ORs) and 95% confidence intervals (CIs) for cognitive dysfunction were estimated for each tertile of dietary hardness by logistic regression analysis, using the lowest category as the reference. Three models were considered in the analysis. Model 1 was adjusted for age (years, continuous variables). In Model 2, we further adjusted for the following potential confounding factors: education (<10, 10–12, or ≥13 years), current employment (yes or no), living alone (yes or no), smoking status (current or past/non-smoking), alcohol consumption status (none, >0 to <46, or ≥46 g/day), habitual exercise (yes or no), BMI (kg/m^2^, continuous), hypertension (yes or no), diabetes (yes or no), depressive symptoms (yes or no), dietary counselling from a doctor or dietitian (yes or no), and energy intake (kcal, continuous) [4,5]. In Model 3, we further adjusted for intake of nutrients, including n-3 PUFA; vitamins A, D, E, B_6_, B_12_, and C; and folate (unit/1000 kcal, continuous) [5,33], which may reduce the risk of cognitive impairment, to consider whether the observed association would be independent of nutrient intake. We tested linear trends using dietary hardness as a continuous variable.

In the sensitivity analysis, we classified participants into halves or quartiles according to dietary hardness or used ≤12 points on the MSP-1100 score as a definition of cognitive dysfunction based on instructions. We repeated the same analysis based on energy-adjusted dietary hardness using total energy intake. Furthermore, we investigated the contribution of each component score of the MSP-1100 (i.e., temporal memory, temporal orientation memory, three-dimensional visual-spatial perception, and short-term memory) based on ordered logistic regression analysis using the highest scores of each component as a reference. The results are presented in terms of beta coefficients and 95% CI. All statistical analyses were performed using SAS version 9.4 (SAS Institute Inc., Cary, NC, USA). All reported *p*-values were two-tailed, and statistical significance was set at *p* < 0.05.

## 3. Results

### 3.1. Association between Dietary Hardness and Selected Characteristics

The mean (SD) age of the participants was 63.5 (3.5) years, and the mean (SD) dietary hardness was 221 (29) mV·s/1000 kcal. The prevalence of cognitive dysfunction assessed using the MSP-1100 among the participants was 7.5% (*n* = 112). Selected characteristics of the participants according to tertiles of energy-adjusted dietary hardness are shown in Table 1. Participants with higher dietary hardness were more likely to be older, have a lower BMI, be unemployed, live with someone, be current smokers, consume alcohol, report habitual exercise and diabetes, and receive dietary counselling from a doctor or a dietitian and less likely to have depressive symptoms than those with lower dietary hardness. Additionally, participants with higher dietary hardness had a higher intake of all the nutrients investigated than those with lower dietary hardness.

### 3.2. Association between Dietary Hardness and the Prevalence of Cognitive Dysfunction and Its Components

Table 2 presents the association between dietary hardness and the prevalence of cognitive dysfunction. The ORs (95% CIs) for cognitive dysfunction in the second and third tertiles of dietary hardness did not significantly differ from those in the first tertile among all models: Model 1: 0.72 (0.45–1.17) and 0.86 (0.54–1.36), Model 2: 0.77 (0.47–1.26) and 0.87 (0.54–1.41), and Model 3: 0.72 (0.43–1.21) and 0.79 (0.43–1.46), respectively (all P for trend ≥ 0.55). We also observed no significant association in the sensitivity analysis based on halves or quartiles of dietary hardness (OR (95% CI) compared with the first category: 0.86 (0.53–1.39) in the second category for halves and 0.80 (0.45–1.42), 0.64 (0.34–1.21), and 1.02 (0.49–2.12) in the second, third, and fourth categories for quartiles in Model 3, respectively) or ≤ 12 points on the MSP-1100 score as a definition of cognitive dysfunction (OR (95% CI): 0.88 (0.37–2.06) and 0.71 (0.24–2.04) in the second and third categories compared with the first category in Model 3, respectively (P for trend = 0.53)). Energy adjustment using total energy intake provided lower dietary hardness (188 ± 29 mV·s/1000 kcal) and no significant associations among all models (ORs (95% CI) for the second and third tertiles compared with the first tertile: Model 1: 0.96 (0.59–1.56) and 1.03 (0.64–1.65), Model 2: 1.06 (0.64–1.75) and 1.17 (0.70–1.94), and Model 3: 1.06 (0.63–1.77) and 1.23 (0.68–2.23), all P for trend ≥ 0.38).

Table 3 shows the association between dietary hardness and each component score of MSP-1100. For temporal orientation, participants in the second tertile (but not those in the third tertile) of dietary hardness had significantly lower score than those in the first tertile among all models. For short-term memory, those in the second tertile of dietary hardness had a significantly higher score than those in the first tertile in Model 1; however, the significant association disappeared after further adjustment for potential confounders (Models 2 and 3).

## 4. Discussion

Contrary to our expectations, we found no evidence of a significant association between dietary hardness and the prevalence of cognitive dysfunction in this cross-sectional study of Japanese men in their 60s. Although we observed some reductions in the ORs for cognitive dysfunction associated with dietary hardness (e.g., OR = 0.87 among those in the third vs. first tertile of dietary hardness after adjustment for socio-demographic factors and OR = 0.79 after further adjustment for protective nutrient intake), these associations did not reach a statistically significant level.

Our study finding that dietary hardness was not associated with cognitive dysfunction was in line with the result reported in the fully adjusted model of Okubo et al. [16] which examined the association between dietary hardness and cognitive function among community-dwelling Japanese men and women aged 69–71 years. More specifically, although Okubo et al. [16] reported that the hardness of the habitual diet was positively associated with cognitive function in a model adjusting for socio-demographic factors, they did not find any evidence of a significant association after adjusting for protective nutrient intake (i.e., the fully adjusted model). Although there was a slight difference in the outcome assessment, the independent association between the hardness of habitual dietary intake and cognitive dysfunction was not observed by Okubo et al. [16] and our participants. Notably, both Okubo et al. [16] and our study estimated dietary hardness in a manner similar to that employed by Murakami et al. [11], who reported a significant association between dietary hardness and waist circumference, minimising the possibility that we did not properly assess the exposure.

Regarding the component score of MSP-1100, our participants in the second tertile of dietary hardness had a significantly lower score of temporal orientation and a significantly higher score of short-term memory compared with those in the first tertile, but the association with only temporal orientation remained statistically significant in the fully adjusted model. Because of these mutually opposite contributions to the total score of MSP-1100, the association between dietary hardness and cognitive dysfunction did not reach a statistically significant level.

There are several possible interpretations of these null findings. First, compared with animals [6,7,8] and humans [9], in which dietary hardness can be defined and changed at the researcher’s discretion, studies in real-life settings, including our study and that of Okubo et al. [16], are challenging. For instance, the hardness of ordinal diets may not be sufficiently high or limited in terms of variation among study populations. In either case, it would be difficult to detect an association between dietary hardness and cognitive function, if it all exists. Second, the null findings could be ascribed to a discrepancy between dietary hardness and masticatory activity. For example, people may have different masticatory activities (frequency of chewing) when eating different foods with the same hardness, thereby widening the gap between dietary hardness and masticatory activity. Third, regarding the relatively young age of the present participants, dietary hardness did not have a sufficient impact on cognitive function among younger individuals with more normal cognitive functions. Its specific threshold affecting cognitive function among different age groups has been unknown owing to the scarcity of studies in this field. Only future studies investigating the association among populations with varying age ranges and a relatively long follow-up period can clarify this issue.

The hardness of food items has been estimated exclusively in Japan; thus, the hardness of diets in other countries and regions is unknown. Given the diversity of food items consumed and the influence of cooking methods [18,19], dietary hardness of habitual dietary intake would vary across countries and regions where people have different dietary cultures. For example, as the moisture content of food items is one of the factors affecting dietary hardness [34,35], it is noteworthy that water intake derived from foods varied between Western countries and Japan (20% [36,37] and 50% [38] of total water intake, respectively). Future studies should estimate the hardness of food items in other countries and regions with different dietary habits and investigate the influence of the hardness of the habitual dietary intake on cognitive function.

The present study has some limitations. First, we estimated dietary hardness based on solid food group intake, which was assessed using the BDHQ. Although the BDHQ is a well-validated dietary questionnaire for estimating selected food and nutrient intake [20,21], no previous study has validated the estimated dietary hardness based on the BDHQ and the database provided by Yanagisawa et al. [19]. Our assumption that the masticatory muscle activity required for consuming particular food items is equivalent between our participants, who were in their 60s, and the reference population among whom the published database was developed [19] might not be valid. In addition, inter-individual variations in the way people cook or digest, which is difficult to measure using the BDHQ, might also affect the estimated dietary hardness. Second, some variables may have helped us better explain the association between dietary hardness and cognitive dysfunction. For example, we had no information on the oral health status of the participants, which can affect both exposure and outcome as a confounder. In addition, we did not collect information on the types of cognitive impairment. Third, we assessed cognitive dysfunction using the MSP-1100, a computerised test battery [24], rather than well-established screening tools, such as the Mini-Mental State Examination [39] and Montreal Cognitive Assessment [40], to maximise the feasibility of this survey in a semi-clinical setting. Therefore, these findings should be interpreted with caution. Fourth, the small number of participants with cognitive dysfunction (*n* = 112) reported in our study might have limited our statistical power to detect the association; the point estimates among those with higher dietary hardness trended in the hypothesised direction. Fifth, the cross-sectional nature of the present study did not permit the assessment of causality, owing to the uncertain temporal order of exposure and outcome. Finally, the present participants were recruited from among those who underwent a health check-up at a Japanese company; thus, the generalisability of the present findings could be limited. Examining the association between the hardness of diet and cognitive dysfunction in other countries might facilitate our understanding of the association, given the wide variation in the hardness of habitual diet due to the diversity of food items consumed and cooking methods across countries.

## 5. Conclusions

Contrary to our expectations, hardness of the habitual diet was not associated with the prevalence of cognitive dysfunction among Japanese men in their 60s, irrespective of adjustment for socio-demographic variables and nutrient intake. Future prospective studies with a relatively long-study period are required to investigate the association between dietary hardness estimated using a validated questionnaire and cognitive dysfunction.

## Figures and Tables

**Table 1:** Selected characteristics of old Japanese men according to tertile of energy-adjusted dietary hardness ^a^.

	Dietary Hardness					
	T1 (*n* = 498)		T2 (*n* = 498)		T3 (*n* = 498)	
Dietary hardness (mV·s/1000 kcal), mean ± SD ^b^	192	13	219	6	253	21
Age (years), mean ± SD	63.2	3.5	63.5	3.5	63.8	3.5
BMI (kg/m^2^), mean ± SD	24.5	3.4	24.2	3.2	23.9	2.8
Education (years)						
<10	18	(3.6)	26	(5.2)	25	(5.0)
10 to 12	275	(55.2)	238	(47.8)	256	(51.4)
≥13	205	(41.2)	234	(47.0)	217	(43.6)
Current employment	359	(72.1)	356	(71.5)	351	(70.5)
Living alone	38	(7.6)	35	(7.0)	30	(6.0)
Current smoker	107	(21.5)	104	(20.9)	119	(23.9)
Alcohol consumption (g/day)						
None	147	(29.5)	125	(25.1)	99	(19.9)
>0 to <46	318	(63.9)	328	(65.9)	337	(67.7)
≥46	33	(6.6)	45	(9.0)	62	(12.4)
Habitual exercise	216	(43.4)	224	(45.0)	289	(58.0)
Hypertension ^c^	192	(38.6)	183	(36.7)	192	(38.6)
Diabetes ^d^	105	(21.1)	94	(18.9)	128	(25.7)
Depressive symptoms ^e^	76	(15.3)	71	(14.3)	43	(8.6)
Dietary counselling	21	(4.2)	24	(4.8)	44	(8.8)
Energy intake (kcal)						
Nutrient intake ^f^	1960	518	1969	588	1957	550
n-3 PUFA (g/1000 kcal)	1.3	0.4	1.4	0.4	1.5	0.5
Vitamin A (μg RE/1000 kcal)	325	301	345	202	456	355
Vitamin D (μg/1000 kcal)	5.6	2.9	7.0	3.5	9.1	5.4
Vitamin E (mg/1000 kcal)	3.4	0.9	3.5	0.9	4.0	1.1
Vitamin B_6_ (mg/1000 kcal)	0.56	0.12	0.64	0.12	0.76	0.16
Vitamin B_12_ (μg/1000 kcal)	4.2	1.9	5.0	2.2	6.1	3.2
Folate (μg/1000 kcal)	144	42	167	42	214	67
Vitamin C (mg/1000 kcal)	43	18	52	19	69	29

BMI, body math index, PUFA, polyunsaturated fatty acids; RE, retinol equivalent; SD, standard deviation; T, tertile. ^a^ Values are n (%), unless otherwise stated. ^b^ Presented as a value per 1000 kcal of energy intake from solid foods (i.e., foods and seasonings). ^c^ Defined as present when participants with SBP ≥ 140 mmHg and/or DBP ≥ 90 mmHg, and/or self-reported medication use for hypertension. ^d^ Defined as present when participants with FPG ≥ 126 mg/dL and/or HbA1c ≥ 6.5%, and/or self-reported medication use for hypertension. ^e^ Defined as present when participants had the short Japanese version of the Center for Epidemiologic Studies Depressive Scale score ≥ 9 [32]. ^f^ Presented as a value per 1000 kcal of total energy intake.

**Table 2:** ORs (95%CIs) for cognitive dysfunction according to tertile of energy-adjusted dietary hardness.

	Dietary Hardness				
	T1 (*n* = 498)		T2 (*n* = 498)	T3 (*n* = 498)	P for Trend ^a^
Cognitive dysfunction, *n* (%) ^b^	42	(8.2)	32 (6.4)	38 (7.6)	
Model 1 ^c^	1	(reference)	0.72 (0.45 1.17)	0.86 (0.54 1.36)	0.55
Model 2 ^d^	1	(reference)	0.77 (0.47 1.26)	0.87 (0.54 1.41)	0.73
Model 3 ^e^	1	(reference)	0.72 (0.43 1.21)	0.79 (0.43 1.46)	0.57

CIs: confidence intervals; ORs: odd ratios; T, tertile. ^a^ Logistic regression model was performed with energy-adjusted dietary hardness as a continuous variable. ^b^ Participants who scored ≤ 13 points on the MSP-1100 were defined as having a cognitive dysfunction [24,26]. ^c^ Adjusted for age (years, continuous). ^d^ Adjusted for variables in Model 1 and BMI (kg/m^2^, continuous), education (<10, 10–12, or ≥13 years), current employment (yes or no), living alone (yes or no), smoking (current or past/non-smoking), alcohol consumption (none, >0 to <46, or ≥46 g/day), habitual exercise (yes or no), hypertension (yes or no), diabetes (yes or no), depressive symptoms (yes or no), dietary counselling (yes or no), and energy intake (kcal, continuous). ^e^ Adjusted for variables in Model 2 and energy-adjusted intakes of n-3 PUFA, vitamins A, D, E, B_6_, B_12_, and C, and folate (unit/1000 kcal, continuous).

**Table 3:** Beta coefficients (95% CIs) for each component of cognitive screening test according to dietary hardness.

	Dietary Hardness					
	T1 (*n* = 498) ^a^		T2 (*n* = 498) ^a^		T3 (*n* = 498) ^a^	
Temporal memory ^b^						
Mean ± SD	2.97	0.17	2.98	0.13	2.99	0.11
Model 1 ^c^	reference		0.32	(−0.55 1.20)	0.75	(−0.24 1.74)
Model 2 ^d^	reference		0.23	(−0.66 1.12)	0.74	(−0.28 1.76)
Model 3 ^e^	reference		0.15	(−0.80 1.10)	0.91	(−0.48 2.30)
Temporal orientation ^b^						
Mean ± SD	3.97	0.17	3.94	0.23	3.97	0.17
Model 1 ^c^	reference		−0.70	(−1.35 −0.04)	−0.02	(−0.77 0.72)
Model 2 ^d^	reference		−0.72	(−1.39 −0.05)	0.12	(−0.64 0.89)
Model 3 ^e^	reference		−0.72	(−1.42 −0.01)	0.30	(−0.64 1.25)
Three-dimensional visual-spatial perception ^b^						
Mean ± SD	1.83	0.38	1.84	0.39	1.80	0.43
Model 1 ^c^	reference		0.09	(−0.25 0.43)	−0.10	(−0.43 0.23)
Model 2 ^d^	reference		0.05	(−0.30 0.40)	−0.15	(−0.50 0.20)
Model 3 ^e^	reference		0.13	(−0.24 0.50)	−0.04	(−0.48 0.40)
Short-term memory ^b^						
Mean ± SD	5.83	0.64	5.90	0.51	5.86	0.55
Model 1 ^c^	reference		0.58	(0.04 1.13)	0.24	(−0.25 0.74)
Model 2 ^d^	reference		0.52	(−0.04 1.08)	0.23	(−0.29 0.75)
Model 3 ^e^	reference		0.54	(−0.05 1.13)	0.19	(−0.47 0.85)

CIs: confidence intervals; SD, standard deviation; T, tertile. ^a^ Ordered logistic regression analysis was performed using the highest scores in each component as references. ^b^ Scores ranged from 0 to 3 for the temporal memory test, from 0 to 4 for the temporal orientation test, from 0 to 2 for the three-dimensional visual-spinal test, and from 0 to 6 for the short-term memory test from the MSP-1100 [24,26]. ^c^ Adjusted for age (years, continuous). ^d^ Adjusted for variables in Model 1 and BMI (kg/m^2^, continuous), education (<10, 10–12, or ≥13 years), current employment (yes or no), living alone (yes or no), smoking (current or past/non-smoking), alcohol consumption (none, >0 to <46, or ≥46 g/day), habitual exercise (yes or no), hypertension (yes or no), diabetes (yes or no), depressive symptoms (yes or no), dietary counselling (yes or no), and energy intake (kcal, continuous). ^e^ Adjusted for variables in Model 2 and energy-adjusted intakes of n-3PUFA, vitamins A, D, E, B_6_, B_12_, and C and folate (unit/1000 kcal, continuous).

## Data Availability

The data are not publicly available but are available upon reasonable request to the corresponding author (fujiwaraay-tky@umin.ac.jp).
